# Usual source and better quality of primary care are associated with lower loneliness scores: a cross-sectional study

**DOI:** 10.1093/fampra/cmad049

**Published:** 2023-04-28

**Authors:** Makoto Kaneko, Satoru Shinoda, Izumi Nakayama, Juan Xu, Susumu Yagome, Atsushi Goto

**Affiliations:** Department of Health Data Science, Yokohama City University, Yokohama, Japan; Department of Biostatistics, School of Medicine, Yokohama City University, Yokohama, Japan; Unit of Public Health and Preventive Medicine, School of Medicine, Yokohama, Japan; Department of Endocrinology and Metabolism, Graduate School of Medicine, Yokohama City University, Yokohama, Japan; Integrity Healthcare Co., Ltd., Chuo, Japan; Department of Health Data Science, Yokohama City University, Yokohama, Japan

**Keywords:** family practice, loneliness, primary health care, quality of healthcare

## Abstract

**Background:**

Loneliness is a global issue, and primary care physicians play an important role in assessing and intervening with loneliness. This study aimed to examine the association between having a usual source of care (USC) or a good quality of primary care, and loneliness.

**Methods:**

This cross-sectional study was conducted in Japan in 2022. A total of 6,000 residents were randomly sampled from the general population, aged 20–74 years. The outcome was the total score of the University of California, Los Angeles (UCLA) 3-item loneliness scale. The exposure included USC and the Person-Centered Primary Care Measure (PCPCM), which assesses the quality of primary care. We conducted a linear regression analysis to adjust for age, sex, educational status, annual household income, self-rated health, living status (whether alone or not), and the existence of physical health problems.

**Results:**

Of the 6,000 residents, 1,277 responded to the survey. The median score of the UCLA 3-item loneliness scale was 6.0 and the mean total score of the PCPCM was 2.62. Of the 1,277 individuals, 713 (55.8%) had USC. Having USC was significantly associated with lower scores on the UCLA 3-item loneliness scale; the coefficient was −0.34 (95% confidence interval (CI): −0.57 to −0.12). Also, the total PCPCM score was significantly associated with lower loneliness scores; the coefficient was −0.56 (*P* < 0.001, 95% CI: −0.78 to −0.35).

**Conclusions:**

Having USC and a better quality primary care were associated with a lower loneliness score. The quality of primary care could be a factor to mitigate patient loneliness.

**Key messages**

- Having usual source of care and a better quality primary care were associated with a lower loneliness score.
- This is the first study to demonstrate that a better quality of primary care is associated with lower loneliness scores.
- The quality of primary care could be a factor to mitigate patient loneliness.

## Introduction

Loneliness is a global health concern. It is defined as a subjective experience where one feels a discrepancy between their actual and desired levels of social relationships.^1^ Recent meta-analyses have revealed that the prevalence of loneliness is 5.3% in young adults, 6.9% in middle-aged adults, and 5.2%–21.3% in older adults worldwide.^2^ Loneliness affects multiple patient aspects, including physical, mental and cognitive health, and mortality.^3–5^ To reduce loneliness, social skills, support, contact, and cognition are important.^6^

Primary care physicians play an important role in assessing and intervening in loneliness.^7^ In primary care settings, physicians can offer opportunities for social contact in their facilities, or link the patients with community resources or other healthcare facilities.^7^ Moreover, while building a good patient–physician relationship, physicians need to know patients’ backgrounds, understand them holistically, and advocate for them.^8^ These experiences of human connection may lessen patients’ loneliness. Therefore, high-quality primary care may reduce loneliness. However, the association between quality of primary care and loneliness remains unclear.

Therefore, this study aimed to examine having a usual source of care (USC) or the quality of care, and loneliness. We hypothesized that better primary care would be associated with lower loneliness. The results can help establish the role of primary care physicians in preventing loneliness and reducing their burden.

## METHODS

### Design

This was a cross-sectional study.

### Setting and participants

The present study is a part of a population-based cross-sectional study to investigate immunity against COVID-19, performed from 30 January to 28 February 2022.^9^ A total of 6,000 residents in the city of Yokohama were randomly sampled from a Japanese population aged 20–74 years on 1 February 2022. Among them, 1,277 individuals (546 men; 731 women) responded to the survey. They provided written informed consent to participate in the study. The city of Yokohama is the most populated basic municipality in Japan and is located next to Tokyo. The population of the city of Yokohama was 3.76 million at the time of 1 February 2022.^10^

**Fig. 1.**
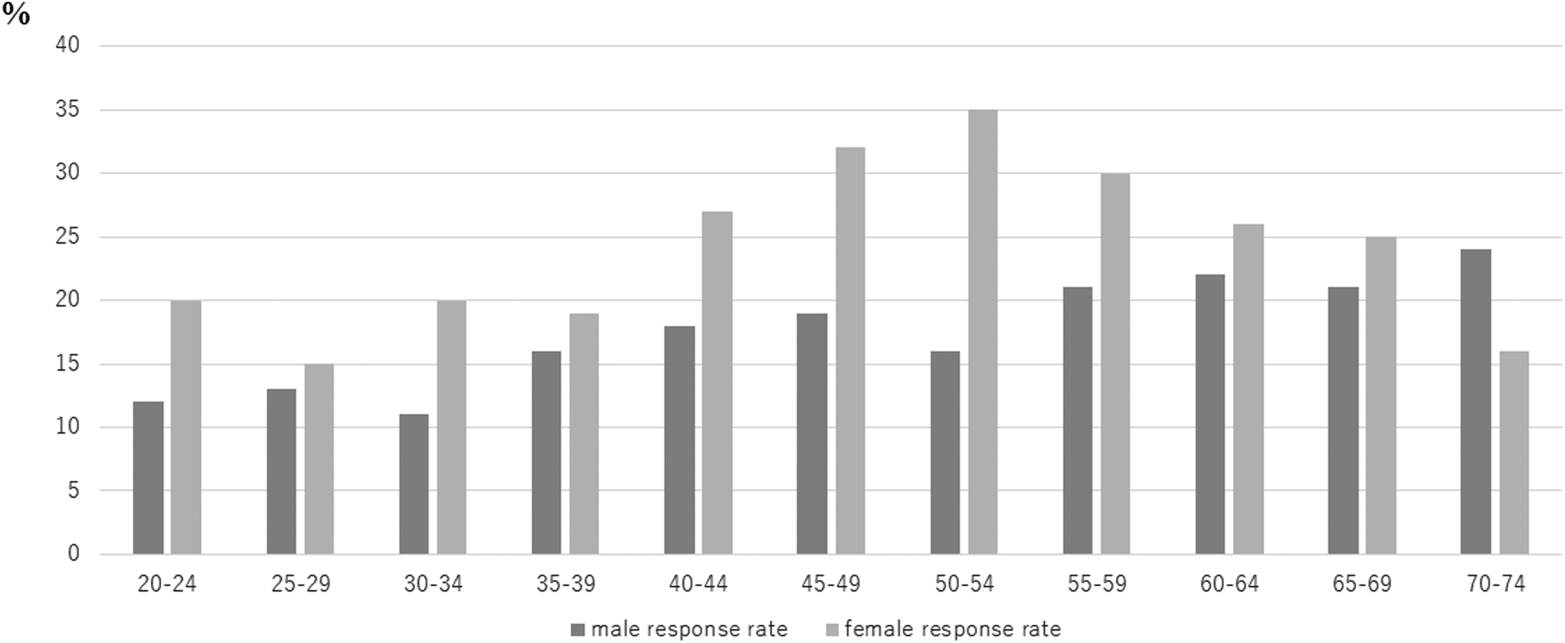
Proportion of the population and the response rate in each age group.

The participants were recruited by post and answered the survey on the web using Microsoft Forms. Figure 1 describes the response rate in each age group and Supplementary Fig. S1a and S1b show the proportion of each age group. In terms of the basic characteristics of the respondents, the response rate by 5-year age groups and sex revealed that the peak response rate in males was in the 70–74 years age group, with a rate of 24%. The peak in females was in the 50–54 years age group, and the rate was 35%.

### Measures

#### Loneliness

We employed the Japanese version of the University of California, Los Angeles (UCLA) 3-item loneliness scale to assess the degree of loneliness among the participants.^11^ The questionnaire comprises the following 3 questions:

1) How often do you feel that you lack companionship?
2) How often do you feel left out?
3) How often do you feel isolated from others?

Respondents answered questions on a four-point Likert scale (1 = Never; 4 = Always). The UCLA Loneliness Scale has been widely utilized worldwide,^2,5,12^ and its Japanese version showed adequate reliability and validity.^11^ Because there is no clear cut-off point, we summed the scores of all answers and used it as a dependent variable (minimum score = 3; maximum score = 12). In addition, based on the previous meta-analysis, we employed the total score of 6 as the cut-off in a sensitivity analysis.^2^

#### Quality of primary care

This study used the Japanese version of the Person-Centered Primary Care Measure (PCPCM) to assess the quality of primary care.^13^ The PCPCM was developed in the United States in 2019 to assess patients’ experience in primary care settings.^14^ It has been translated into 28 languages.^15^ Patient experience is a crucial indicator of the quality of primary care,^16^ and previous studies have reported that social isolation is negatively associated with patient experience.^17^ This measure assesses 11 important domains of primary care using 11 items: accessibility, comprehensiveness, integration, coordination, relationship, continuity, advocacy, family context, community context, goal-oriented care, and health promotion. Because the role of primary care physicians is ambiguous in Japan,^18^ the Japanese version of the PCPCM used the question, “Is there a medical facility to whom you usually go if you are sick or need advice about your health?” to identify whether the participant had a USC.^13,19^The study used a binominal variable (USC) and the PCPCM score (continuous variable: 1–4) as independent variables. In the model which examined the association between the PCPCM score and the UCLA loneliness scale, we only analysed the participants who had USC.

#### Covariates

Covariates were determined by known associations with loneliness and patient experience based on previous literature.^3,17^ We included age (categorical variable; 5-year age group), sex (binary variable), education status (categorical variable: less than high school, high school, junior college, and more than or equal to college), annual household income (categorical variable: <3, 3–5.99, 6–8.99, 9–11.99, 12–14.99, and ≥15 million JPY), self-rated health (categorical variable; excellent, good, neutral, poor, and very poor), living status (binary variable: alone or not), and the existence of physical health problems (binary variable). Physical health problems included cancer, heart disease, stroke, diabetes, hypertension, dyslipidemia, asthma, chronic obstructive pulmonary disease, chronic kidney disease, chronic hepatitis, immunodeficiency, and hematological disorders. All variables were collected from the participants’ self-reports, except for age and sex.

#### Statistical analyses

**Fig. 2.**
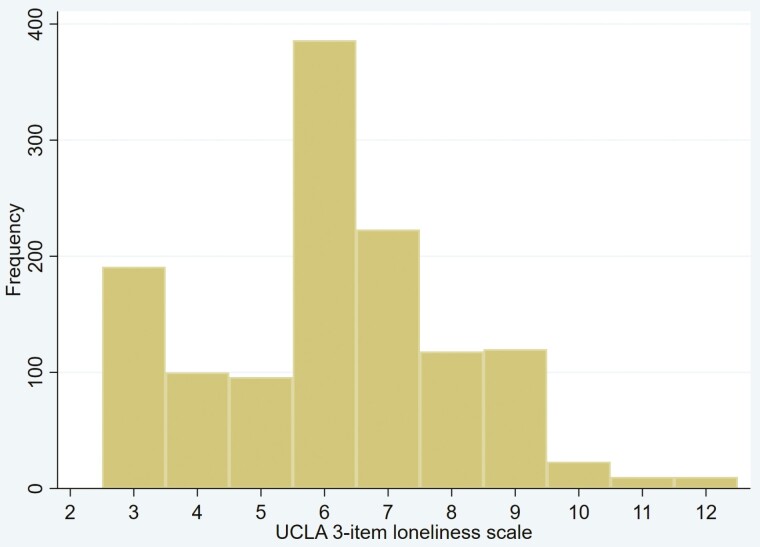
Distribution of the UCLA 3-item loneliness scale.

Continuous variables were presented as mean and standard deviation or median and interquartile range. Categorical variables were presented as numbers and proportions. In Table 1, the difference between a group with USC and without USC is tested by Chi-square test for categorical variables and by Student’s *t*-test for continuous variables. Figure 2 shows a histogram of the UCLA 3-item loneliness scale. We also performed the analysis in 2 models using multivariable linear regression analysis to adjust for covariates.

**Table 1.** Demographics of the participants (*n* = 1,277)

	Overall *n* (%)	With USC *n* = 713 *n* (%)	Without USC *n* = 564 *n* (%)	*P*-value
Sex Male Female	731 (57.2) 546 (42.8)	418 (58.6) 295 (41.4)	313 (55.5) 251 (44.5)	0.262
Age (year) 20–24 25–29 30–34 35–39 40–44 45–49 50–54 55–59 60–64 65–69 70–74	69 (5.4) 68 (5.3) 73 (5.7) 89 (7.0) 125 (9.8) 170 (13.3) 197 (15.4) 152 (11.9) 116 (9.1) 106 (8.3) 112 (8.8)	34 (4.8) 29 (4.1) 24 (3.4) 41 (5.8) 49 (6.9) 79 (11.1) 106 (14.9) 94 (13.2) 74 (10.4) 85 (11.9) 98 (13.7)	35 (6.2) 39 (6.9) 49 (8.7) 48 (8.5) 76 (13.5) 91 (16.1) 91 (16.1) 58 (10.3) 42 (7.4) 21 (3.7) 14 (2.5)	<0.001
Education Less than high school High school Junior college More than or equal to college Others	12 (0.9) 294 (23.0) 355 (27.8) 611 (47.8) 5 (0.4)	8 (1.1) 186 (26.1) 195 (27.3) 322 (45.2) 2 (0.3)	4 (0.7) 108 (19.1) 160 (28.4) 289 (51.2) 3 (0.5)	0.037
Annual household income (million JPY) <3.00 (≒27,000 US dollar) 3.00–5.99 6.00–8.99 9.00–11.99 12.00–14.99 ≥15.00 I don’t know	202 (15.8) 373 (29.2) 256 (20.1) 212 (16.6) 78 (6.1) 58 (4.5) 98 (7.7)	132 (18.5) 212 (29.7) 138 (19.4) 108 (15.1) 46 (6.5) 28 (3.9) 49 (6.9)	70 (12.4) 161 (28.5) 118 (20.9) 104 (18.4) 32 (5.7) 30 (5.3) 49 (8.7)	0.043
Self-rated health status Excellent Good Neutral Poor Very poor	54 (4.2) 464 (36.3) 436 (34.1) 318 (24.9)	4 (0.6) 41 (5.8) 264 (37.0) 233 (32.7) 171 (24.0)	1 (0.2) 13 (2.3) 200 (35.5) 203 (36.0) 147 (26.1)	0.019
Living alone Yes No	130 (10.2) 1147 (89.8)	54 (7.6) 659 (92.4)	76 (13.5) 488 (86.5)	0.001
Existence of physical health problems Yes No	496 (38.8) 781 (61.2)	385 (54.0) 328 (46.0)	111 (19.7) 453 (80.3)	<0.001
Median of the total score of UCLA loneliness scale (IQR)	6.0 (5.0–7.0)	6.0 (4.0–7.0)	6.0 (5.0–8.0)	
Mean of the total score of UCLA loneliness scale (standard deviation)	6.12 (1.97)	5.92 (1.94)	6.37 (1.97)	<0.001
The total score of UCLA loneliness scale: 6 or more	890 (69.7)	478 (67.0)	412 (73.0)	0.02

USC: usual source of care.

IQR: interquartile range.

UCLA: University of California, Los Angeles.

The difference between a group with USC and without USC are tested by Chi-square test for categorical variables and by Student’s *t*-test for continuous variables.

Model 1: An independent variable was the existence of a binominal variable (USC), and the dependent variable was the score on the UCLA 3-item loneliness scale (continuous variable). The model targeted all the participants.

Model 2: The independent variable was the score on the PCPCM (continuous variable), and the dependent variable was the score on the UCLA 3-item loneliness scale (continuous variable). The model targeted participants with USC because only these participants answered the PCPCM questionnaire.

As a sensitivity analysis, we used a robust standard error to consider the overdispersion of the dependent variable. We checked the assumptions of linear regression, such as normality and homoscedasticity of residuals, and linearity between exposure and outcome by data visualization; the regression diagnostics did not detect violation of the assumptions. However, because the loneliness score was not a continuous variable, we performed a regression analysis with robust standard errors to address a possible incorrect variance function or heteroscedasticity as a sensitivity analysis. Also, we added the sensitivity analysis with each observation weighted by the inverse probability using the distribution of the target population by 5-year age groups and sex to consider the overrepresentation of the sample. In addition, we performed a logistic regression analysis using the cut-off, the total loneliness score of 6.^2^ We also conducted subgroup analysis by sex and age (<65 and ≥65 years) to examine the impact of having USC or quality of primary care for loneliness among different groups. The dataset did not include any missing data. All statistical analyses were performed using StataCorp software, 2017 (Stata Statistical Software: Release 15. College Station, TX, StataCorp LLC).

## RESULTS

Among the 6,000 residents who were randomly sampled, 1,277 individuals (male: 42.8%) responded to the survey. The response rate was 21.3%. The median score of the UCLA three-item loneliness scale was 6.0 (interquartile range: 5.0–7.0) and the proportion of the participants with a total score of 6 or more was 69.7%. Of the 1,277 individuals, 713 (55.8%) had USC. Table 1 shows the demographics of individuals with and without USC. The mean total score of PCPCM was 2.62 (*SD* = 0.66) among individuals with USC. The mean scores for each item in PCPCM were as follows: accessibility: 2.93 (*SD* = 0.74), comprehensiveness: 2.86 (*SD* = 0.73), integration: 2.70 (*SD* = 0.80), coordination: 2.59 (*SD* = 0.83), relationship: 3.18 (*SD* = 0.79), continuity: 2.27 (*SD* = 0.92), advocacy: 2.57 (*SD* = 0.81), family context: 2.26 (*SD* = 0.96), community context: 2.21 (*SD* = 0.90), goal-oriented care: 2.55 (*SD* = 0.85), and health promotion: 2.69 (*SD* = 0.85).

**Fig. 3.**
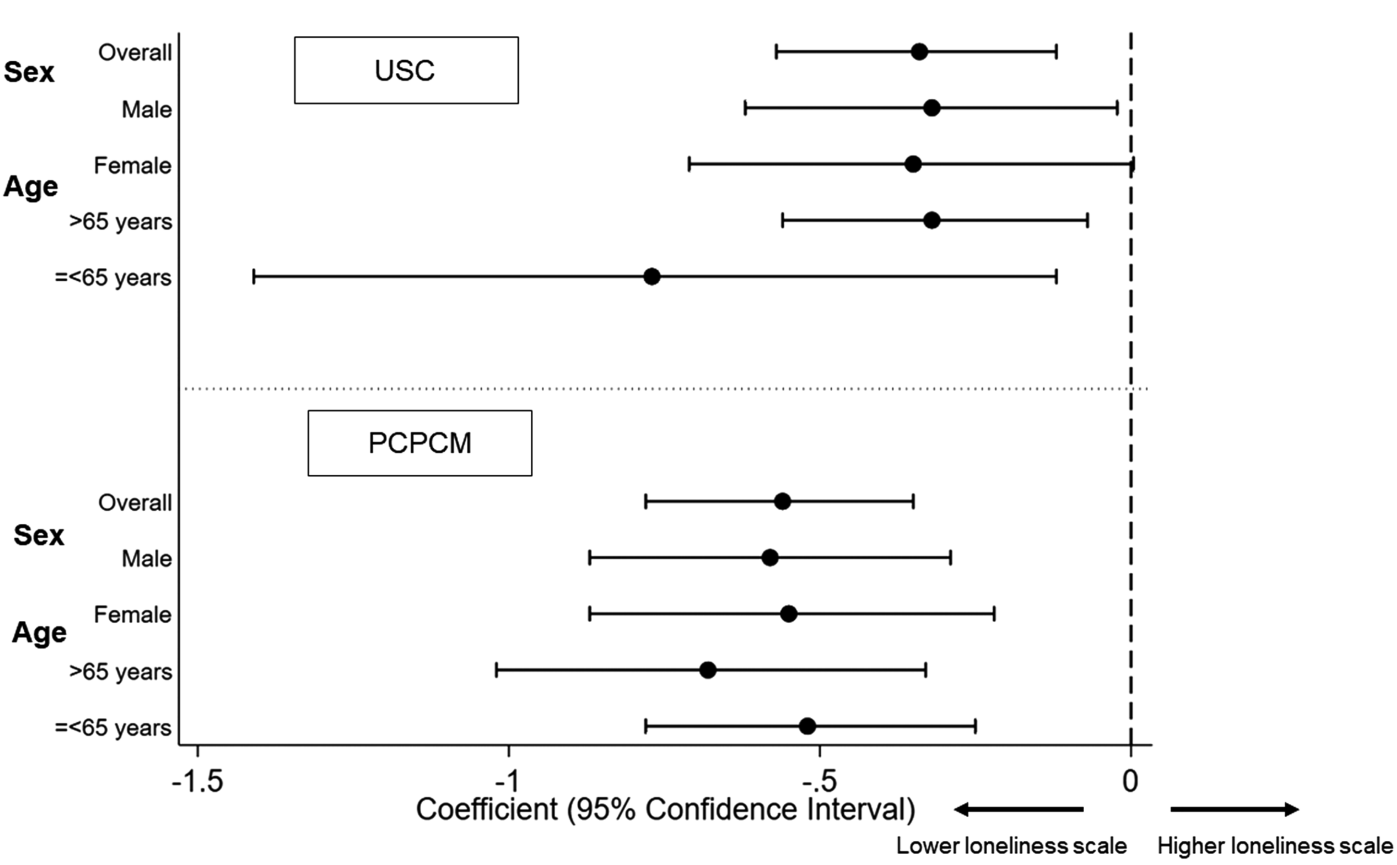
Forest plot of the overall results and subgroup analyses.

Table 2 shows the results of multivariable linear regression analysis and Table 3 describes the results of the crude and adjusted coefficients for loneliness. In model 1, having USC was significantly associated with lower scores on the UCLA 3-item loneliness scale; the coefficient was −0.34 (*P* = 0.003, 95% confidence interval (CI): −0.57 to −0.12). In model 2, the total PCPCM score was significantly associated with lower loneliness scale scores; the coefficient was −0.56 (*P* < 0.001, 95% CI: −0.78 to −0.35). The trends of the results by the inverse probability using the distribution of the target population by 5-year age groups and sex were similar: −0.33 (*P* = 0.011, 95%: −0.59 to −0.08) in model 1 and −0.61 (*P* < 0.001, 95% CI: −0.85 to −0.37) in model 2. In logistic analyses, the odds ratio of having USC was 0.79 (*P* = 0.10, 95% CI: 0.60 to 1.05) and that of the total PCPCM score was 0.48 (*P* < 0.001, 95% CI: 0.36 to 0.63) for the loneliness score of 6 or more. In the sensitivity analysis, the coefficients and 95% CIs were almost the same. Figure 3 shows the forest plot of the results of the main and subgroup analyses. The trends in the results were similar to those of the main analysis. In all sex and age groups, the total PCPCM score was significantly associated with lower loneliness scores.

**Table 2.** Association between the UCLA loneliness scale and each variable: the results of multivariable linear regression

Model 1 (*n* = 1,277)	UCLA loneliness scale Coefficient (95% CI, *P*-value)
Existence of USC	−0.34 (−0.57 to −0.12, *P* = 0.003)
Sex Female Male	Reference −0.067 (−0.29 to 0.16, *P* = 0.56)
Age (year) 20–24 25–29 30–34 35–39 40–44 45–49 50–54 55–59 60–64 65–69 70–74	Reference −0.17 (−0.81 to 0.47, *P* = 0.61) −0.095 (−0.73 to 0.54, *P* = 0.77) −0.15 (−0.75 to 0.46, *P* = 0.63) −0.26 (−0.83 to 0.31, *P* = 0.37) −0.060 (−0.60 to 0.49, *P* = 0.83) −0.012 (−0.52 to 0.49, *P* = 0.96) 0.50 (−1.06 to 0.054, *P* = 0.077) −0.67 (−1.26 to −0.094, *P* = 0.023) −1.03 (−1.62 to −0.44, *P* = 0.001) −1.06 (−1.65 to −0.47, *P* < 0.001)
Education Less than high school High school Junior college More than or equal to college Others	Reference −0.11 (−1.19 to 0.98, *P* = 0.84) 0.087 (−1.00 to 1.17, *P* = 0.86) 0.03 (−1.05 to 1.11, *P* = 0.96) 1.13 (−0.85 to 3.10, *P* = 0.26)
Annual household income (million JPY) <3.00 (≒27,000 US dollar) 3.00–5.99 6.00–8.99 9.00–11.99 12.00–14.99 ≥15.00 I don’t know	Reference −0.24 (−0.56 to 0.086, *P* = 0.15) −0.40 (−0.77 to −0.041, *P* = 0.029) −0.58 (−0.97 to −0.20, *P* = 0.003) −0.53 (−1.04 to −0.02, *P* = 0.004) −0.89 (−1.46 to −0.31, *P* = 0.002) −0.17 (−0.63 to 0.30, *P* = 0.48)
Self-rated health status Very poor Poor Neutral Good Excellent	Reference 1.81 (0.092 to 3.52, *P* = 0.039) 1.44 (−0.20 to 3.09, *P* = 0.086) 0.96 (−0.69 to 2.61, *P* = 0.25) 0.29 (−1.36 to 1.94, *P* = 0.73)
Living alone No Yes	Reference 0.66 (0.30 to 1.01, *P* < 0.001)
Existence of physical health problems No Yes	Reference 0.19 (−0.055 to 0.43, *P* = 0.13)

Model 2 (*n* = 713)	UCLA loneliness scale Coefficient (95% CI, *P*-value)
PCPCM	−0.58 (−0.79 to −0.36, *P* < 0.001)
Sex Female Male	Reference −0.12 (−0.42 to 0.17, *P* = 0.42)
Age (year) 20–24 25–29 30–34 35–39 40–44 45–49 50–54 55–59 60–64 65–69 70–74	Reference 0.52 (−0.42 to 1.46, *P* = 0.28) 0.20 (−0.78 to 1.19, *P* = 0.68) 0.066 (−0.805 to 0.94, *P* = 0.88) −0.29 (−1.14 to 0.56, *P* = 0.51) −0.13 (−0.92 to 0.65, *P* = 0.74) 0.34 (−0.41 to 1.10, *P* = 0.37) 0.33 (−1.10 to 0.44, *P* = 0.40) −0.40 (−1.19 to 0.39, *P* = 0.32) −0.87 (−1.66 to −0.09, *P* = 0.029) −0.79 (−1.55 to −0.023, *P* = 0.043)
Education Less than high school High school Junior college More than or equal to college Others	Reference 0.63 (−0.69 to1.95 *P* = 0.35) 0.67(−0.65 to 2.00, *P* = 0.32) 0.57 (−0.74 to 1.88, *P* = 0.39) 1.98 (−0.93 to 4.90, *P* = 0.18)
Annual household income (million JPY) <3.00 (≒27,000 US dollar) 3.00–5.99 6.00–8.99 9.00–11.99 12.00–14.99 ≥15.00 I don’t know	Reference −0.23 (−0.63 to 0.17, *P* = 0.26) −0.085 (−0.55 to 0.038, *P* = 0.72) −0.46 (−0.69 to 0.028, *P* = 0.064) −0.37 (−1.02 to 0.028, *P* = 0.26) −1.06 (−1.84 to −0.27, *P* = 0.008) −0.27 (−0.89 to 0.36, *P* = 0.40)
Self-rated health status Very poor Poor Neutral Good Excellent	Reference 1.06 (−0.83 to2.96, *P* = 0.27) 1.00 (−0.82 to 2.82, *P* = 0.28) 0.74 (−1.08 to 2.61, *P* = 0.42) 0.0057 (−1.82 to 1.84, *P* = 0.99)
Living alone No Yes	Reference 0.51 (−0.006 to 1.02, *P* = 0.053)
Existence of physical health problems No Yes	Reference 0.0087 (−0.30 to 0.32, *P* = 0.96)

PCPCM: Person-Centered Primary Care Measure.

UCLA: University of California, Los Angeles.

USC: usual source of care.

**Table 3.** Crude and adjusted coefficients for the loneliness score

Independent variable	Crude coefficient (95% CI)	Adjusted coefficient* (95% CI): Linear regression	Adjusted coefficient* (95% CI): Robust standard error
Existence of USC	−0.46 (−0.67 to −0.24)	−0.34 (−0.57 to −0.12)	−0.34 (−0.58 to −0.11)
Total score of the PCPCM (per 1-point increment)	-0.74 (-0.95 to -0.53)	−0.56 (−0.78 to −0.35)	−0.58 (−0.80 to −0.36)

USC: usual source of care.

PCPCM: Person-Centered Primary Care Measure.

CI: confidence interval.

^*^Adjusted for age, sex, years of education, annual household income, self-rated health, living alone or not, and existence of physical health problems.

## DISCUSSION

This study revealed that the existence of USC and a better quality of primary care were associated with lower loneliness scores. These results imply that the quality of primary care could be a factor to mitigate patient loneliness.

To the best of our knowledge, this is the first study to demonstrate that a better quality of primary care is associated with lower loneliness scores. The existing literature has reported that patients experiencing loneliness tend to visit primary care physicians more frequently.^20^ Additionally, primary care physicians can identify and address patients’ loneliness with other health professionals or community resources.^7^ Nevertheless, whether a better quality of primary care is associated with lower loneliness scores has not been evaluated. The potential mechanisms of these results might be explained by the doctor–patient relationship. Understanding a patient holistically and offering appropriate support is a core component of primary care.^8,21^ Such a doctor–patient relationship could mitigate patients’ loneliness.^8^ Additionally, referral and non-referral pathways are possible mechanisms to explain these findings.^7^ In referral pathways, primary care physicians refer patients to other healthcare services^22–24^ and non-healthcare sectors.^23,25–27^ Non-referral pathways include intervention for loneliness in a primary care clinic by the physicians,^28–30^ external agencies,^31^ teams of community health and social care professionals that connect adults who have been discharged to volunteers.^7,32^

The clinical implication of this study is that improving the quality of primary care may be associated with lower loneliness score. Because loneliness is a common problem in primary care settings,^33^ having USC and improving the quality might be important for patients. Moreover, the present study was conducted during the COVID-19 pandemic, and previous studies have suggested that more people experienced loneliness during this time, affecting their mental health and well-being in Japan^34^ as well as other countries.^35,36^ In primary care settings, physicians can screen and address loneliness through in-person consultation and telehealth based on their knowledge of the patient’s medical conditions and life circumstances.^37^ This is an important primary care physician role, and the results of the study might provide evidence for promoting primary care physicians’ intervention for loneliness.

### Strengths

This is the first study to demonstrate that a better quality of primary care is associated with lower loneliness scores. This study employed random sampling among the general public to ensure internal validity. We targeted a wide range of generations (20–74 years old) to extrapolate the results not only for older adults but also for the younger generation. Moreover, in this study, we assessed the comprehensive quality of primary care using PCPCM. Because the results of the subgroup analysis demonstrated similar trends to those of the main analysis, we suggested no interaction between sex and age.

### Limitations

This study has some limitations. First, based on the cross-sectional nature of the study, we cannot determine the causality between the quality of primary care and loneliness. Some socially isolated participants might not have obtained enough information to find USC and could not build a better relationship with their primary care physicians.^38^ Additionally, regarding the UCLA 3-item loneliness scale, because the minimal clinically important difference remains unclear, the difference which we detected needs to be interpreted with caution. Second, we could not adjust for mental health problems because our questionnaire did not include items regarding mental health issues. Mental health problems can affect both the quality of primary care from a patient’s perspective and their experience of loneliness. We were also unable to take into account the social support that the participants received. Social support might reduce loneliness.^39^ Thus, social support could be a confounder between having USC and loneliness. However, our study also showed that, among the participants who had USC, better primary care was associated with lower loneliness scores. As social support may not be directly associated with the quality of primary care, the association between social support and the quality of primary care may be valid. Third, the response rate was relatively high among older people in males and middle-aged people in females. Also, COVID-19 might increase the prevalence of loneliness more than usual. This could affect the results. However, the results of the sensitivity analysis by the inverse probability using the distribution of the target population by 5-year age groups and sex showed a similar trend. Fourth, the study was conducted in an urban city in Japan. Therefore, the results should be interpreted carefully when generalizing them.

## CONCLUSIONS

This study demonstrated that having USC and a better quality of primary care are associated with lower loneliness scale scores. The quality of primary care could be a factor to mitigate patient loneliness.

## Supplementary material

Supplementary material is available at *Family Practice* online.

cmad049_suppl_Supplementary_Figure_S1a

cmad049_suppl_Supplementary_Figure_S1b

cmad049_suppl_Supplementary_Checklist

## Data Availability

The data underlying this article will be shared on reasonable request to the corresponding author. [scolor_start auto][fcolor_start auto]5 (0.4)[/fcolor][/scolor]

## Transparency

All authors had full access to all the data (including statistical reports and tables) in the study and take responsibility for the integrity and accuracy of the data analyses. The lead author affirms that the manuscript is reliable and accurate and provides a transparent account of the study being reported, that no important aspects of the study have been omitted, and that any discrepancies from the study as planned have been explained.
