# Supplementary figures and images for: Usual source and better quality of primary care are associated with lower loneliness scores: a cross-sectional study
Source: Fam Pract. 2023 Apr 28;41(3):312–20. doi: 10.1093/fampra/cmad049 (PMC11167986; doi:10.1093/fampra/cmad049)

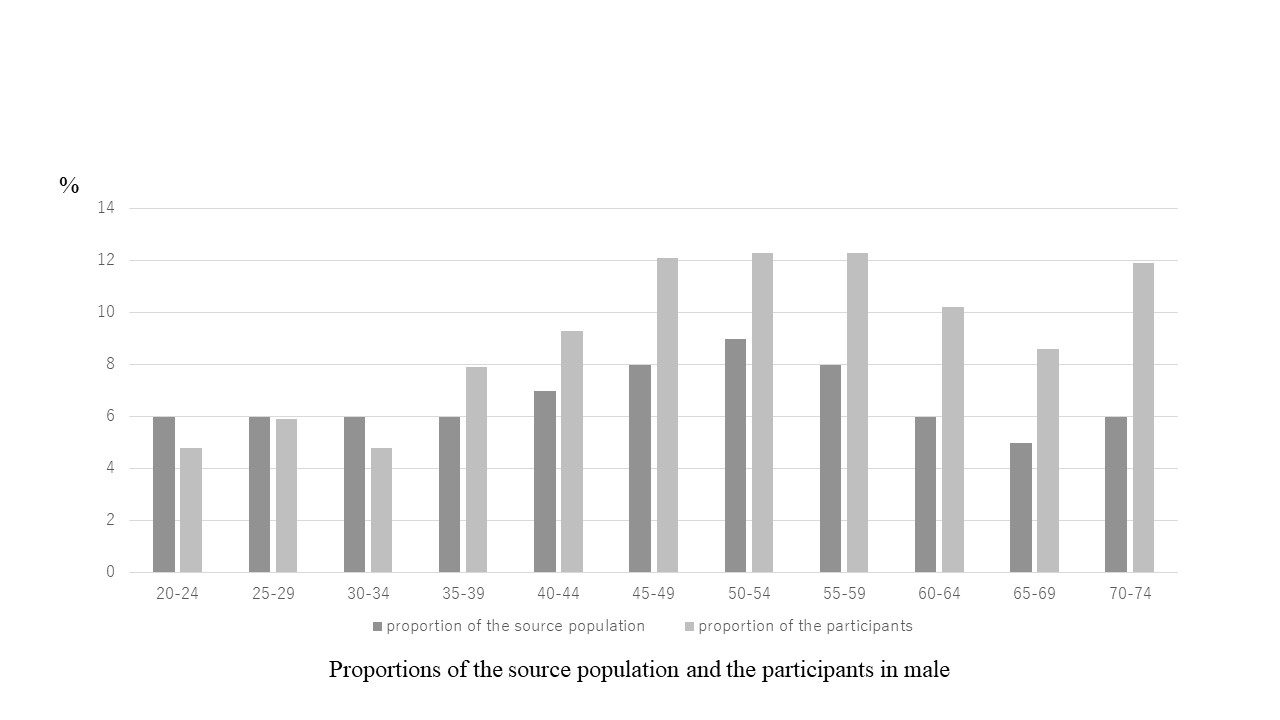

Supplement: cmad049_suppl_Supplementary_Figure_S1a [file cmad049_suppl_supplementary_figure_s1a.jpeg]

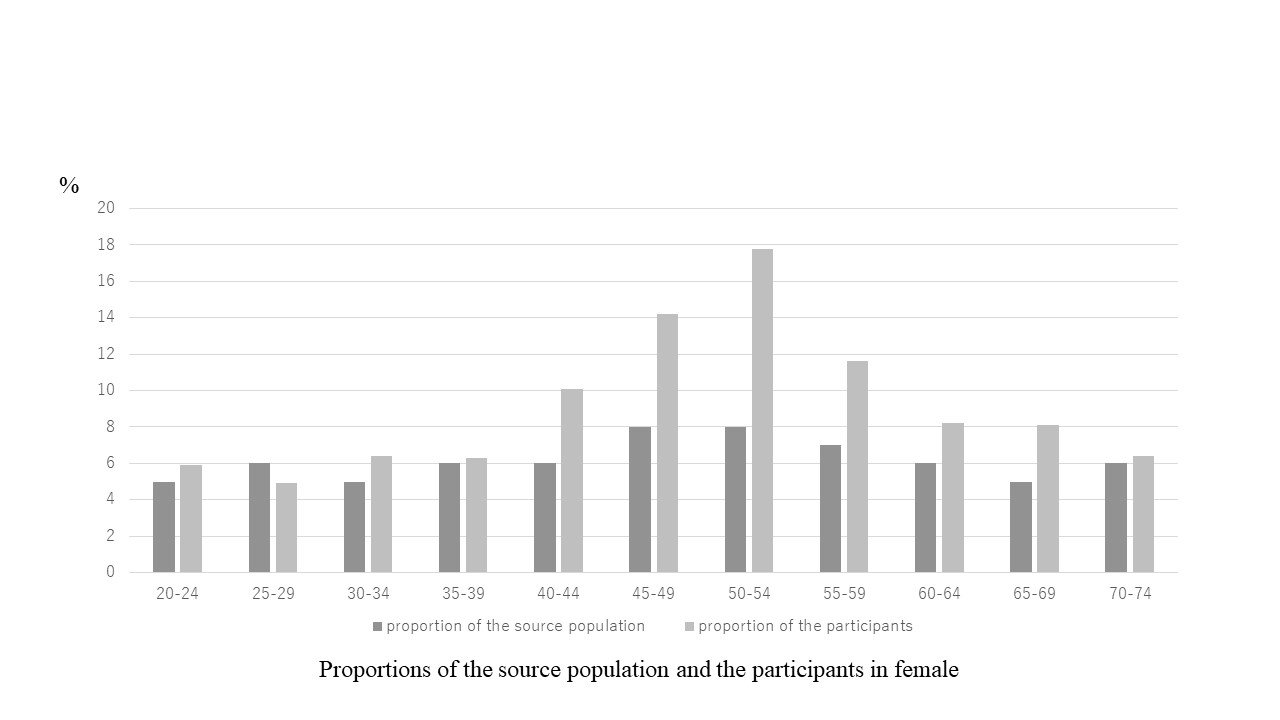

Supplement: cmad049_suppl_Supplementary_Figure_S1b [file cmad049_suppl_supplementary_figure_s1b.jpeg]
